# Pet Grief: Tools to Assess Owners’ Bereavement and Veterinary Communication Skills

**DOI:** 10.3390/ani9020067

**Published:** 2019-02-21

**Authors:** Ines Testoni, Loriana De Cataldo, Lucia Ronconi, Elisa Silvia Colombo, Cinzia Stefanini, Barbara Dal Zotto, Adriano Zamperini

**Affiliations:** 1 Department of Philosophy, Sociology, Education and Applied Psychology (FISPPA), University of Padova, 35131 Padua, Italy; decataldolory@virgilio.it (L.D.C.); l.ronconi@unipd.it (L.R.); elisasilvia.colombo@yahoo.it (E.S.C.); stef.cinzia@gmail.com (C.S.); dalzotto.barbara@gmail.com (B.D.Z.); adriano.zamperini@unipd.it (A.Z.); 2 Sagol Creative Arts Therapies Research Center, Haifa 31905, Israel

**Keywords:** veterinary counseling, pet grief, veterinary communication skills

## Abstract

**Simple Summary:**

In Italy, there are approximately 60.5 million companion animals, and 92% of Italian owners consider them as family members. Despite the growing interest in pet bereavement and in end-of-life (EOL) issues in veterinary medicine across the world, there are still very few Italian studies on the psychological impact of losing a pet, and there are no instruments in the Italian language to assess grief, following the death of a companion animal and the impact of effective veterinary communication skills on pet bereavement. The aim of this study is the Italian adaptation of instruments (Pet Bereavement Questionnaire (PBQ); Regret of Bereaved Family Members (RBFM); Shared Decision-Making Questionnaire (SDM-Q-9); Consultation and Relational Empathy Measure (CARE)), which can be useful in assessing bereavement in companion animal owners and communication skills in veterinary medicine. All the instruments obtained good internal reliability. The results showed that the use of the Italian versions of all the instruments is useful, and that the CARE, the SDM-Q-9, and the Regret of Bereaved Family Members (RBFM) developed for the human healthcare context, may also be used in veterinary medicine, and in further Italian pet bereavement and EOL veterinary studies.

**Abstract:**

In Italy, there are still very few studies on the psychological impact of losing a pet. The need to fill this gap springs from the fact that pet loss counseling services are increasingly being activated. The aim of this study is the Italian adaptation of instruments for veterinary counseling services. The survey instruments adapted were: Pet Bereavement Questionnaire (PBQ) to describe the individual experience of pet-grief; Regret of Bereaved Family Members (RBFM) to assess the family regret; Shared Decision-Making Questionnaire (SDM-Q-9) for decision making in end of life; Consultation and Relational Empathy Measure (CARE) to assess the veterinarian relational empathy during clinical encounters. All the instruments obtained good internal reliability, and the results of the confirmative factor analysis of all the Italian versions were in accordance with the original ones. The correlational analysis among the variables evidenced the following aspects: the more the owner feels involved by the veterinarian in the decision making process the more the veterinarian is perceived by the owner as empathetic; when the veterinarian is perceived as empathic and the decision making is shared the owners’ pet bereavement distress and regrets are reduced; negative dimensions of bereavement (grief, guilt, anger, intrusive thoughts and decisional regrets) are strictly linked to each other, therefore if one dimension increases or decreases the others do too. The path analysis suggests that developing a veterinary relationship-centered care practice may be beneficial for pet owners facing end-of-life issues and the death of their companion animals since it showed that shared-decision making strategies and empathic communication may reduce negative dimensions of bereavement that may complicate grief. Interestingly, adopting shared decision-making strategies may contribute to be perceived as more empathic. These aspects may be taken into consideration in end-of -life communication training in veterinary medicine.

## 1. Introduction

Grief and mourning counseling is a form of psychological support that aims to help people cope with loss. This form of suffering is often brought about by the death of a loved one or another significant loss, including the death of a pet. Indeed, companion animals are an important part of family life, and some studies consider them as attachment figures whose loss elicits a significant grief response [1], one similar to the mourning caused by the death of relatives [2,3,4]. Western social culture, unfortunately, still disenfranchises pet-grief, so it is difficult to support mourners [5,6]. Pet bereavement is a field in which grief counseling may be very useful. This kind of mourning attracts the support of others, but the cultural disenfranchising of this form of suffering may generate negative consequences due to the lack of social support. When this happens, people withdraw from their networks, feeling helpless and experiencing strong regrets or guilt, and grief can remain unresolved, thereby leading to depression [7,8,9]. In these situations, counseling may provide an avenue for healthy resolution, and research in this area has been slowly growing [10,11]. 

Psychological counseling is useful in providing insights into the decision making and coping processes for chronic illness, end-of-life (EOL) care and mourning [10,12,13,14]. Veterinary EOL care has unique characteristics, as it ought to tackle peculiar challenges because of the widespread disenfranchising attitudes towards the suffering of pets and their owners. The possible denial of the sorrow caused by the death of pets by veterinarians does not help to overcome mourning of the owners, but exacerbates the extent of the distress. In particular, the relational and communication skills of veterinarians and their way of providing treatment to pets may alleviate or aggravate the grief of the owner [15].

Pets are unable to verbalize needs and wishes, that is why their EOL care and decision-making ability are medically, emotionally, and ethically challenging to all the actors involved in a relationship with them and may require competent counseling intervention to support owner grief [12]. The counselor could improve the development of a collaborative plan between veterinarian and the owner, recognizing the implications of this kind of loss [10], helping both veterinarians and mourners by improving their positive relationships [16]. Since communication skills play a crucial role in the veterinary profession [17], counselors could help to improve this competence. In particular, they might support the process of sharing the care death plan, which help to reduce mourners’ regrets: “what ifs?”, “if onlys” [18,19,20,21]. Furthermore, empathetic communication is the hallmark of effective client support during EOL treatment and after a patient’s death [16]. 

Although, a growing body of research shows the importance of this area of study, there are still very few Italian studies on the psychological impact of losing a companion animal. The present assessment is intended to contribute towards filling this gap, offering some instruments in the Italian language that can be very useful in the development of this field. 

The importance of preparing an adequate assessment of all these factors springs from the fact that pet loss counseling services have begun to be activated in all the Western countries and in Italy, where there are approximately 60.5 million pets [22]. Since 92% of people living with a companion animal, in this country, believe they could not live without them, as they are considered part of their family and a source of wellbeing [23], we deem it helpful to adapt instruments for assessing this kind of mourning and its relationship with the veterinarian. 

The purpose of the research was to adapt and translate into the Italian language the psychometric properties of instruments for assessing, on the one hand, the psychological impact of pet grief and on the other hand, to determine the effectiveness of veterinary communication. In particular, for the variable “pet grief”, we checked two dimensions. The first one was inherent in the psychological impact of losing a pet, utilizing the Pet Bereavement Questionnaire (PBQ) [24] and hypothesizing that its dimensions are in sync with Italian sensitiveness. The second one was the intensity of regret felt by mourners assessed by the Regret of Bereaved Family Members (RBFM) [25] which is usually employed to evaluate the decisions on palliative care in human medicine. For the veterinarian relational abilities, the Consultation and Relational Empathy Measure (CARE) [26] is usually utilized to measure the quality of the relationships between healthcare professionals and their human patients. Adapting all the items to the pet condition, we hypothesized that it adequately fits into the Italian context. To measure the process of shared decision making in clinical encounters from the patients’ perspective, the nine-item Shared Decision-Making Questionnaire (SDM-Q-9) [27], usually used in human medicine was selected, hypothesizing that it can also be adaptable in the Italian veterinarian context. 

Veterinary EOL care should focus on maximizing patient comfort and minimizing suffering while providing a collaborative and supportive partnership with the caregiver client; empathetic communication is the hallmark of effective client support during EOL treatment and after a patient’s death [16]. It has been proposed that, as in human medicine, the optimal model for the veterinarian-client relationships is the relationship-centered care, characterized as a partnership in which negotiation and shred decision making between the veterinarian and the client are used to incorporate into all aspects of care the client’s perspectives and the recognition of the role animals play in the life of the client [28]. The relationship centered process intends to support the receipt of goal concordant care [29], which is vital in veterinary medicine, specifically as it relates to end-of-life choices [30], since it has been shown that the perception of the professional support provided by the veterinarian contributes to client’s grief [31]. In this process, empathy has been highlighted as a core communication skill [15,16,17,32]. In human medicine, research has identified effective communication as one of the most important contributors of high-quality end-of-life care [33,34], and healthcare resulting in bereaved caregivers feeling more anxious, depressed, traumatized, or regretful may reflect poor-quality EOL care and communication [35,36]. Although research is needed to determine if effective communication may have the same positive impact in veterinary medicine as it does in human medicine [30], the authors hypothesize that effective communication between veterinarians and clients is likely to affect owner’s bereavement. Learning from the human medical profession regarding serious illness communication is essential to provide high quality veterinary care in a goal-concordant way, and mental health professionals play a key role in this learning process, and its application in veterinary medicine [30].

First of all, we aspired to verify whether this assessment structure, aimed at measuring the factors mentioned above, confirms the importance of the role of the veterinarian in the management of the owners’ mourning. This verification requires not only the validation of appropriate questionnaires, but also the calculation of their correlations and the measurement of the impact that the different variables have on the negative feelings of grief. The ultimate goal of our survey is to provide a tool that can be utilized, on the one hand, by counselors and veterinarians who want to support mourners and, on the other, by researchers who intend to develop this area of studies. 

## 2. Hypotheses, Participants, Material and Methods

The fundamental hypotheses of our studies were the following: (1) The instruments for assessing the perceived relational empathy of the veterinarian, the decision making process, bereavement including guilt, grief and anger, family regret are suitable for the Italian population of pet owners; (2) Guilt, grief, anger intrusive thoughts and decisional regrets are minimized when the veterinarian is empathic and the decision making is shared; (3) Decisional regrets and intrusive thoughts increase with grief, guilt and anger; (4) Time (age, time of relationship between owner and pet, age of the pet) is inversely correlated with negative emotions of bereavement and regret; (5) Gender differences and level of studies are inversely correlated with negative emotions of bereavement and regret. (6) In particular, we wanted to verify the role of shared decision making and the perceived empathy of veterinary, hypothesizing that they can reduce significantly anger, guilt, intrusive thoughts and regret in decision making.

Participants were selected through Facebook. An invitation with a link to the survey was posted on the social media platform, explaining that it was a research project fostered by the University of Padova. The inclusion criteria were as follows: having experienced the death of a pet, being at least 21 years old, having coped with the illness and EOL issues with the help of the veterinarian, and understanding the Italian language. Data were collected from July 2017 to December 2017. Among the 377 pet owners who responded to the questionnaire, 354 (318 females and 36 males) provided analyzable data. Participants completed the self-administered online survey. Their age ranged from 21 to 80 years (32% of participants were aged 21–34 years; 25% 35; 25% 45–54; 13% 55–64; 4% 65–74; 1% 74–80). Most respondents had experienced the death of dogs (61%) and cats (35%) (other animals are 4%) in the last 2 years (55%). The mean age of the pets at death was 11 years (SD = 6). The average length of pet ownership was 10 years (SD = 5). The median age of the pets at death was 12 years (interquartile range = 7). The median length of pet ownership was 11 years (interquartile range = 8). With respect to the SD of the age of the pet at death and the average length of pet ownership, Skewness and kurtosis are near 0 for both variables (skewness: −0.21 and −0.16; kurtosis: −0.27 and −0.82, respectively, for age of the pet at death and for length of pet ownership) but Shapiro normality test is significant (*p* < 0.001), so these data are not normally distributed. The questionnaire (see Appendix A for questionnaire) was introduced by an information sheet that included a general description of the survey and a consent form ensuring anonymity and privacy. 

The socio-demographic part assessed participants’ characteristics (age, gender, educational level, companion animal’s characteristics, including species, time since companion animal’s death, ownership duration), and euthanasia-related issues. The instruments and their psychometric characteristics were as follows:

- The Consultation and Relational Empathy Measure (CARE) [26] to assess the owners’ perception of the veterinarian’s relational empathy. It is a five-point Likert Scale ranging from poor to excellent, useful for measuring the quality of healthcare professionals’ relationships, based on empathy, capability to perceive the pet conditions and its feelings and to care for it [37]. The total scoring was obtained by adding all the items’ values (min 10, max 50). It has a high internal consistency (Cronbach’s alpha > 0.9). The CARE has been extensively validated and it is widely used to measure the interpersonal quality of healthcare encounters in human medicine [38]. The permission to slightly change the wording of the questionnaire to fit into the veterinary context and translation of the questionnaire was provided by the author (2, May, 2017) (i.e., we changed: “How was the doctor at…” To “How was the veterinary at…”; “Being Positive … having a positive approach and a positive attitude; being honest but not negative about your problems” to “Being Positive … having a positive approach and a positive attitude; being honest but not negative about your dog’s/cat’s problems”).
- The Shared Decision-Making Questionnaire (SDM-Q-9) [27] is a nine-item instrument to assess the owners’ perceived level of involvement in shared decision-making related to the treatment and care of pets. It is a brief self-assessment instrument for measuring the process of shared decision-making in clinical encounters in human medicine, that is, the patients’ perceived level of involvement in decision-making related to their own treatment and care [27]. To use the questionnaire in the veterinary healthcare context, the wording of the SDM-Q-9 was slightly changed, focusing it on the pet’s condition and the owners. The instrument was developed according to the following definition: SDM is defined as an interactive process in which both parties (patient and physician) are equally and actively involved and share information to reach an agreement for which they are jointly responsible [39]. The SDM-Q-9 is unidimensional and shows high internal consistency (Cronbach’s alpha > 0.9) as well as high item discrimination, both indicating the high reliability of the instrument. The nine statements are rated on a six-point scale from “completely disagree” to “completely agree”. Scores of all items should be added (total score max 45, min 0). The permission to slightly change the wording of the questionnaire to fit into the veterinary context and translation of the questionnaire was provided by the authors (9, May, 2017) (i.e., we changed “My doctor made clear that a decision needs to be made” to “My veterinarian made clear that a decision needs to be made”; “My doctor told me that there are different options for treating my medical condition” to “My veterinarian told me that there are different options for treating the medical condition of my dog/cat”).
- To assess the pet grief distress, we utilized Pet Bereavement Questionnaire (PBQ) [24], a 16-item four-point Likert scale composed of three distinct factors: Grief (items: 2, 3, 5, 7, 10, 12, 15), Anger (items: 1, 4, 11, 13, 14), and Guilt (items 6, 8, 9, 16). The Pet Bereavement Questionnaire (PBQ) was developed to fill the need for a brief, acceptable, well-validated instrument for use in studies of the psychological impact of losing a pet (Hunt and Padilla, 2006). The PBQ has been proven to have good internal reliability (Cronbach’s alpha = 0.87), good factor structure, and good construct validity. The permission to translate the questionnaire into the Italian language was provided by the authors (13, July, 2012).
- To assess the owner’s intensity of regret, we utilized the Regret of Bereaved Family Members (RBFM) [25], a seven-item scale on a five-point self-reported Likert scale for bereaved family members measuring their self-evaluation on the decision to admit their loved ones to palliative care units. The wording of the RBFM was changed to re-focus it on the veterinary healthcare context. RBFM measures three aspects: overall degree of regret (total scoring), evaluation of decisional regret (items 1,2,3,4), and severity of intrusive thoughts about regret (items 5,6,7). The internal consistency was high for both “intrusive thoughts of regret” (Cronbach’s alpha = 0.85) and “decisional regret” (Cronbach’s alpha = 0.79). The permission to slightly change the instructions of the questionnaire to adapt it to the veterinary context and to translate it into the Italian language was provided by the authors (5, May, 2017).
- The ontological representations of death were measured with the Testoni Death Representation Scale (TDRS; Testoni, Ancona, & Ronconi, 2015), which is a monofactorial scale composed by 6 items on a 5-point Likert scale. The TDRS explores the destiny of the personal identity of the responders between death as total annihilation and as a passage.

All instruments, except for TDRS, have been forward- and backward-translated by two independent translators. The final version of the translation was produced by all the members of the research group.

First, we explored item response distributions of the four questionnaires (CARE, SDM, PBQ and RBFM) and we performed a Confirmatory Factor Analysis (CFA), hypothesizing items in the same construct as in the original version of each questionnaire. Given the ordinal nature of the data, we used the Diagonally Weighted Least Squares (DWLS) robust estimator. Several fit indices were considered to evaluate the models: Comparative Fit Index (CFI), Tucker-Lewis Index (TLI), Root-Mean-Square Error of Approximation (RMSEA), and Weighted Root Mean Square Residual (WRMR). Cut-off values for fit were considered adequate if CFI and TLI were >0.90, RMSEA less than 0.06 [40] and WRMR was less than 1.0 [41]. Reliability of all construct scales was also evaluated by Cronbach’s alpha. 

Second, we examined correlations between constructs and with participants’ and companion animal’s characteristics and, third, we evaluated the effects of a shared decision-making, related to pet’s treatment and care, and veterinarian’s relational empathy on psychological distress for losing a pet by a path analysis, using Maximum Likelihood estimator method. Analyses were carried out using SPSS 24 (IBM, Armonk, NY, USA) for descriptive statistics, reliability of all questionnaires and correlations, and using the R package lavaan [42] for CFAs and path analysis.

The study followed the American Psychological Association (APA) Ethical Principles of Psychologists and Code of Conduct and the principles of the Declaration of Helsinki. Furthermore, it was approved by the Ethics Committee of the University of Padova (Ethical Code CCDFB2584D2F02096722F5DCB035CDDD). Participants were informed about the study’s aims and procedures and assured that participation was voluntary. The confidentiality of their responses was guaranteed. Informed consent was obtained from all participants.

## 3. Results

Percentages: Fifty-nine percent of participants opted for euthanasia and 88% were present during the procedure. In their opinion, euthanasia was performed at the right time (91%), the veterinarian was sensitive toward the owner and the pet during euthanasia (96%) and provided proper information on the procedure (95%).

Confirmatory factor analysis: Most of the items showed a left-skewed distribution (skewness ranging between −1.02 and −0.57 for all items of CARE; between −1.17 and −0.57 for seven items of SDM; between −1.46 and −0.62 for six items of PBQ; between −1.27 and −0.48 for three items of RBFM), and some item showed a right-skewed distribution (skewness ranging between 0.61 and 2.35 for eight items of PBQ; between 0.73 and 2.07 for four items of RBFM), further supporting the appropriateness of an analytical approach that takes the ordinal nature of the data into account [43].Confirmatory factor analysis results indicated a good fit of the hypothesized factor structure for CARE, RBFM, SDM and a lower fit, but always adequate for PBQ (Table 1).

Factor loadings were significant at the 0.001 level in all CFA models. Standardized coefficients ranging between 0.90 and 0.96 with the average factor loading of 0.93 for CARE; ranging between 0.50 and 0.93 with the average factor loading of 0.79 for SDM; ranging between 0.27 and 0.83 with the average factor loading of 0.60 for PBQ; ranging between 0.59 and 0.98 with the average factor loading of 0.76 for RBFM. Reliability indices were good for all instruments, with Cronbach’s alpha coefficients ranging between 0.60 and 0.98 (see Table 2).

Correlations: The correlational analyses among the variables of the study (Table 2) evidenced in particular the positive correlation between SDM and CARE; PBQ Anger, Grief, Guilt and Total score, which in turn are positively correlated with RBFM Intrusive Thoughts and Decisional Regret. Negative correlations emerged among CARE with all the variables of PBQ and RBFM, which in turn are negatively correlated with SDM total score as well. The positive correlations among the demographic characteristics of the owners and those of the pets with the variables of the study were the following: CARE and SDM with owner’s age, pet’s age at death and years of relationship with owner, pet’s euthanasia (Table 3). The most important negative correlations were the followings: owners’ age with PBQ Anger, Guilt and total score; Gender with PBQ Grief, Anger and total score; Educational level with PBQ Grief, Anger and total score, and all RBFM dimensions; having Children with PBQ Guilt and total score; Time from pet’s death with PBQ Grief and Total score, RBFM Intrusive thoughts and TDRS; Pet’s age at death with PBQ Anger, Guilt and Total score, RBFM Decisional Regrets and total score; Duration of the relationship with PBQ Anger, Guilt and total score, RBFM Intrusive thoughts and Decisional regrets; Pet’s Euthanasia with PBQ Anger and Guilt, RBFM Decisional regrets and total score.

**Figure 1:**
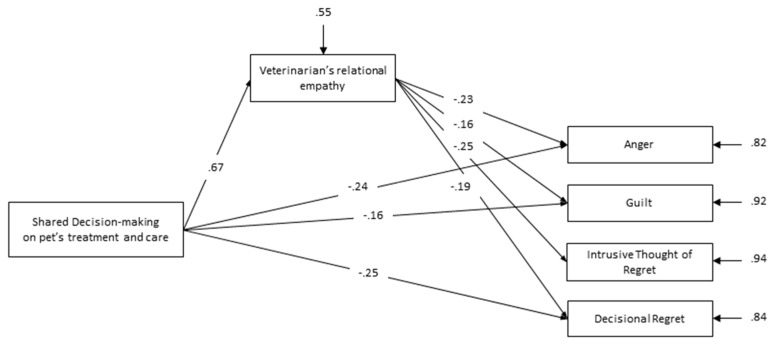
Path analytic model of the effects of shared decision-making related to pet’s treatment and care and veterinarian’s relational empathy on psychological distress for losing a pet (Coefficients are standardized structural coefficients).

Path model: We started with a full model having all direct effects from shared decision-making and veterinarian’s relational empathy on all measures of psychological distress for losing a pet considered in our study, except for Grief of PBQ. In fact, correlations examination attests to the absence of association of shared decision-making and veterinarian’s relational empathy with grief. In the full model, there was only one non-significant effect, the direct effect of shared decision-making on intrusive thoughts of regret. Thereafter, we eliminated this non-significant effect to get a final model, which include only the significant relationships between the variables at the 0.05 level (Figure 1). The estimated model showed an adequate fit to the data, as indicated by the following fit indexes: χ2(1, *n* = 327) = 0.00, *p* = 0.958, CFI = 1.00, TLI = 1.02, RMSEA = 0.00, WRMR = 0.01.

The whole model accounted for 18% of the variance for Anger, 8% of the variance for Guilt, 50% of the variance for total bereavement distress, 6% of the variance for Intrusive Thoughts of Regret, 16% of the variance for Decisional Regret and 45% of the variance for veterinarian’s relational empathy. Results of the Sobel test supported a mediating role of veterinarian’s relational empathy in links between shared decision-making and psychological distress for losing a pet (β = −15, z = −3.44, *p* = 0.001 for Anger; β = −11, z = −2.24, *p* = 0.025 for Guilt; β = −17, z = −4.52, *p* < 0.001 for Intrusive Thoughts of Regret; β = −0.13, z = −2.89, *p* = 0.004 for Decisional Regret).

## 4. Discussion

Our study focused on the validation of instruments suitable for improving pet grief counseling research and strategies to help cope with mourning and adjusting to the loss of a companion animal. This form of counseling could be a service offered along with palliative care and euthanasia plans in veterinarian clinics to improve the relationships between veterinarians and owners and to facilitate the elaboration of mourning after the death of a pet. 

The reason for advancing the development in this field was due to the growing demand all over the world for questionnaires that assesses pet grief, relationship with veterinarians and the effectiveness of palliative care with companion animals across countries and languages, while Italian questionnaires on such issues have been rare. This article summarizes the global research and discusses the empirical evidence on this issue. The hypotheses of the research were all confirmed, and the validation operation showed that their translation into the Italian language is possible and useful. Indeed, the battery used in the present research, composed of CARE, SDM-Q-9 and RBFM, made it easy to adapt to EOL and the pet grief field. The psychometric characteristics of the Italian version of all the instruments were investigated by evaluating their construct validity and reliability (consistency). The internal consistency is good, as shown by the fact that the Cronbach alpha values are above the threshold of acceptability for the scales calculated according to the English version and for those calculated based on the results of the Italian sample. Factorial analysis of the main components showed that their factor structure was appropriate for the Italian sample. These instruments can be useful in studies focused on the quality of the relationships between veterinarians, owners and pets in palliative care settings, but it could also be utilized to study the euthanasia decision making processes and administering further instruments. Each specific instrument can be utilized autonomously and integrated with other scales of measurement. The further development of the research with this population could improve the analysis of relationships and differences among the variables we considered.

Furthermore, the analyses showed how the negative dimensions of bereavement are strictly linked to each other: guilt, grief, anger intrusive thoughts and decisional regrets. On the one hand, our study evidenced that all these variables, which normally typify complicated grief in human loss, characterize also pet loss. On the other hand, it showed that when the veterinary is empathic and the decision making is shared such negative effect of pet loss can be significantly reduced. This result confirms that the way veterinarians communicate with their clients greatly influences how owners can cope with their grief. If the veterinary is able to support grievers, helping them to understand the condition of their pet and its suffering, the negative effect of mourning decreases. Therefore, this result suggests the potential benefit of utilizing an inter-professional care team to develop a veterinary relationship-centered care practices. As literature widely shows [44,45], grief counseling is really helpful in managing loss. It has been increasingly used in order to help mourners to handle their loss experience. Usually, in different ways, bereaved people when sharing suffering with their counselors, they tell the stories of their losses and consciously examine their painful feelings, becoming more reconciled with the idea that their relationships with the deceased are no more possible or cannot be the same as in the past. Through this process, they can reinterpret or remember aspects of their histories with loved ones and find resilient strategies. Thanks to the counseling treatment, they are empowered to come to terms with their losses in a healthy way and to reinterpret their life into hopeful stories for their futures [46,47].

Mental health professionals may play an important role in the care team increasing EOL communication training for all the veterinary staff, and supporting owners not only after the death but also during the final phase of their companion animal’s lives. However, the study highlights that time in all its forms can mitigate the negative effect of mourning. As literature has widely evidenced in the area of the so called complicated grief [48,49], most people are able to adjust to loss over time.

Indeed, the age of owners and of the pet and the time of their relationship alleviate regret, guilt and anger. In our opinion, all these variables should be taken into account in veterinary clinics, and a counseling service can help in providing better support to animal owners.

Men seemed to cope easier than women and to deal with the death of their pet with minor collateral effects, as literature on human grief and mourning already described [48]. However, this result could be linked to the small number of male participants and to the high percentage of women. To better analyze this circumstance, future developments could be oriented to balance the percentage of men, as literature has already shown gender differences in mourning pain. In particular, it was pointed out that men tend to mask emotional affection behavior and underreport psychological issues and the strength of attachment for their companion animals [50], and, since the sampling of this research was done through voluntary participation, thanks to the Internet, the small male presence partly confirms this figure, as reported already in the literature.

## 5. Conclusions

The aim of the study is to translate and adapt into the Italian language instruments for assessing the owners’ grief following the death of their companion animals and the impact of effective veterinary communication skills on bereavement.

The majority of Italian pet owners consider their companion animals as family members, and, as their death may elicit significant grief responses similar to the one caused by the death of a beloved person, social support must be available. The activation of pet loss counseling services is even more important if we consider that pet bereavement is a form of disenfranchised grief that may cause a lack of social support. Furthermore, counseling may also be useful in providing insights into the decision making and coping processes for chronic illness, EOL care and mourning. 

In Italy there is currently a growing awareness of the need of giving greater attention to pet bereavement and end-of-life issues in veterinary medicine given the important role that companion animals play in our families. Pet loss counseling services are starting to be activated but there are no instruments in the Italian language to assess grief following the death of a companion animal and the impact of effective veterinary communication skills on pet bereavement. 

The aim of this study is to contribute to filling this gap in order to help the development of pet loss counseling services and encourage Italian scientific research in this field. The findings suggest that the Italian version of the Pet Bereavement Questionnaire can be useful to assess pet bereavement distress in Italian owners, and that the CARE, SDM-Q-9 and RBFM adapted to the veterinary context and translated into the Italian language may be useful to assess effective communication skills in veterinary medicine. 

## Figures and Tables

**Table 1:** Fit indices of Confirmatory Factor Analysis for each questionnaire.

Questionnaire	N	Factors	Chi-square	df	*p*	CFI ^5^	TLI ^6^	RMSEA [90% CI] ^7^	WRMR ^8^
CARE ^1^	343	1	5.022	35	1	1	1	0.000 [0.000–0.000]	0.302
SDM ^2^	316	1	52.914	27	0.002	0.994	0.992	0.055 [0.033–0.077]	1.084
PBQ ^3^	327	3	281.02	101	<0.001	0.953	0.944	0.074 [0.064–0.084]	1.437
RBFM ^4^	338	2	17.852	13	0.163	0.995	0.992	0.033 [0.000–0.068]	0.798

Notes: ^1^ CARE = Consultation and Relational Empathy, ^2^ SDM = Shared Decision-Making Questionnaire, ^3^ PBQ = Pet Bereavement Questionnaire, ^4^ RBFM = Regret of Bereaved Family Members, ^5^ CFI = Comparative Fit Index, ^6^ TLI = Tucker-Lewis Index, ^7^ RMSEA = Root Mean Square of Approximation and 90% CI = Confidence Interval at 90% level, ^8^ WRMR = Weighted Root Mean Square Residual.

**Table 2:** Descriptive statistics (Range, Mean, Standard Deviation, Cronbach’s alpha) and correlations for study variables

Variables	Range	M	SD	alpha	1	2	3	4	5	6	7	8	9
1. CARE ^1^ Total score	10–50	38.10	12.52	0.98									
2. SDM ^2^ Total score	0–45	29.40	13.55	0.94	0.67 ***								
3. PBQ ^3^ Grief	0–21	14.66	4.93	0.87	0.01	-0.03							
4. PBQ ^3^ Anger	0–15	3.52	3.10	0.60	−0.38 ***	−0.39 ***	0.49 ***						
5. PBQ ^3^ Guilt	0-12	4.68	3.63	0.75	−0.25 ***	−0.27 ***	0.40 ***	0.57 ***					
6. PBQ ^3^ Total score	0–48	22.87	9.43	0.86	−0.21 ***	−0.25 ***	0.84 ***	0.80 ***	0.78 ***				
7 RBFM ^4^ Intrusive Thoughts of Regrets	3–5	5.70	3.24	0.77	−0.22 ***	−0.17 **	0.29 ***	0.50 ***	0.45 ***	0.49 ***			
8. RBFM ^4^ Decisional Regrets	4–20	7.95	4.48	0.86	−0.34 ***	−0.38 ***	0.07	0.41 ***	0.39 ***	0.32 ***	0.50 ***		
9. RBFM ^4^ Total score	7–35	13.64	6.71	0.85	−0.33 ***	−0.33 ***	0.19 ***	0.51 ***	0.48 ***	0.45 ***	0.82 ***	0.91 ***	
10. TDRS ^5^ Total score	6–30	18.01	6.81	0.83	0.02	−0.01	−0.03	−0.02	0.02	−0.002	−0.04	−0.01	−0.03

Notes: ^1^ CARE = Consultation and Relational Empathy, ^2^ SDM = Shared Decision-Making Questionnaire, ^3^ PBQ = Pet Bereavement Questionnaire, ^4^ RBFM = Regret of Bereaved Family Members, ^5^ TDRS = Testoni Death Representation Scale, * *p* < 0.05; ** *p* < 0.01; *** *p* < 0.001.

**Table 3:** Correlation of study variables with participants’ characteristics and companion animal’s characteristics.

Participant’s and Pet’s Characteristics	CARE ^1^ Total	SDM ^2^ Total	PBQ ^3^ Grief	PBQ ^3^ Anger	PBQ ^3^ Guilt	PBQ ^3^ Total	RBFM ^4^ Intrusive Thoughts of Regrets	RBFM ^4^ Decisional Regrets	RBFM ^4^ Total	TDRS ^5^ Total
Participants’ characteristics										
Age (year)	0.20 ***	0.17 **	−0.03	−0.17 **	−0.26 ***	−0.17 **	−0.06	−0.06	−0.07	0.05
Gender ^a^	0.04	0.07	−0.19 ***	−0.12 *	−0.08	−0.17 **	−0.06	−0.04	−0.05	0.05
Educational level ^b^	0.01	−0.07	−0.15 **	−0.11 *	−0.08	−0.15 **	−0.18 **	−0.16 **	−0.19 ***	0.00
Work ^c^	0.06	0.07	−0.05	−0.18 **	−0.24 ***	−0.18 **	−0.24 ***	−0.16 **	−0.22 ***	0.01
Children ^d^	0.09	0.07	−0.06	−0.09	−0.17 **	−0.13 *	−0.04	0.05	0.01	0.04
Live alone ^d^	0.04	0.09	−0.03	−0.01	−0.09	−0.05	−0.10	−0.08	−0.10	−0.10
Pets’ characteristics										
Animal species ^e^	−0.04	0.00	0.05	0.04	0.06	0.06	0.07	−0.01	0.03	0.04
Time from pet’s death (year)	−0.14 **	−0.13 *	−0.25 ***	0.03	0.03	−0.11 *	−0.18 **	0.09	−0.03	−0.12 *
Pet’s age at death (year)	0.19 ***	0.23 ***	−0.05	−0.18 **	−0.19 ***	−0.16 **	−0.10	−0.25 ***	−0.21 ***	0.02
Pet’s Ownership duration (year)	0.16 **	0.19 ***	−0.01	−0.18 **	−0.18 **	−0.13 *	−0.11	−0.17 **	−0.16 **	0.02
Pet’s euthanasia ^d^	0.20 ***	0.31 ***	0.05	−0.13 *	−0.19 ***	−0.09	−0.07	−0.31 ***	−0.24 ***	0.04

Notes: ^a^ Coded 0 = female 1 = male, ^b^ Coded 0 = none 1 = elementary school 2 = middle school 3 = professional school 4 = high school 5 = degree 6 = postgraduate, ^c^ Coded 0 = no worker 1 = part-time worker 2 = full-time worker, ^d^ Coded 0 = no 1 = yes, ^e^ Coded 0 = Cat 1 = Dog, ^1^ CARE = Consultation and Relational Empathy, ^2^ SDM = Shared Decision-Making Questionnaire, ^3^ PBQ = Pet Bereavement Questionnaire, ^4^ RBFM = Regret of Bereaved Family Members, ^5^ TDRS = Testoni Death Representation Scale, * *p* < 0.05; ** *p* < 0.01; *** *p* < 0.001.

## Appendix A

### Appendix A.1. Consultation and Relational Empathy Measure (CARE)

Le chiediamo di ripensare agli ultimi colloqui che ha avuto con il suo medico veterinario nell’ultimo periodo di vita del suo animale da compagnia e di dare una valutazione del suo medico veterinario relativamente ai seguenti aspetti su una scala da 1 a 5.

1 = scarso 2 = sufficiente 3 = buono 4 = molto buono 5 = eccellente

Come valuterebbe il suo medico veterinario relativamente ai seguenti aspetti:

1. Metterla a suo agio… (essere amichevole e cordiale nei suoi confronti, trattarla con rispetto; non in modo freddo o brusco).	**1**	**2**	**3**	**4**	**5**
2. Lasciarle raccontare la sua “storia”… (darle il tempo di descrivere dettagliatamente la malattia del suo animale da compagnia con le sue parole, senza interromperla o sviarla).	1	2	3	4	5
3. Ascoltare veramente… (prestare grande attenzione a quello che lei stava dicendo; non guardare gli appunti o il computer mentre lei stava parlando).	1	2	3	4	5
4. Essere interessato a lei come persona nella sua totalità… (chiedere/sapere dettagli rilevanti della sua vita, della sua situazione; non trattarla “solo come un numero”).	1	2	3	4	5
5. Comprendere pienamente le sue preoccupazioni… (comunicare di aver accuratamente compreso le sue preoccupazioni, senza trascurare o ignorare nulla).	1	2	3	4	5
6. Dimostrare cura e compassione… (sembrare veramente interessato, connettersi con lei sul piano umano; non essere indifferente o “distaccato”).	1	2	3	4	5
7. Essere positivo… (avere un approccio positivo e un atteggiamento positivo; essere onesto, ma non negativo riguardo ai problemi del suo animale da compagnia).	1	2	3	4	5
8. Spiegare le cose chiaramente… (rispondere esaustivamente alle sue domande, spiegare in modo comprensibile, dare adeguate informazioni; non essere vago).	1	2	3	4	5
9. Aiutarla a prendere il controllo della situazione… (esplorare insieme a lei quello che lei può fare da solo per migliorare la salute del suo animale da compagnia; incoraggiarla piuttosto che “darle delle lezioni”).	1	2	3	4	5
10. Fare con lei un piano di azione… (discutere le opzioni, coinvolgerla nelle decisioni nella misura in cui vuole essere coinvolto/a; non ignorare il suo punto di vista).	1	2	3	4	5

### Appendix A.2. Shared Decision-Making Questionnaire (SDM-Q-9)

Di seguito troverà delle affermazioni che si riferiscono alle decisioni riguardo il trattamento del problema di salute del suo animale da compagnia.

Con il termine “trattamento” non intendiamo soltanto le cure mediche in senso stretto, ma tutte le diverse possibili opzioni che, su proposta del medico veterinario, i proprietari possono trovarsi a dover prendere in considerazione(es. proseguimento delle cure mediche, cure palliative, interruzione delle cure in attesa della morte naturale, eutanasia, etc.).

Per favore barri per ogni affermazione la casella più appropriata per lei. Faccia sempre riferimento alla decisione sul trattamento che è stata presa durante gli ultimi incontri con il medico veterinario.

Per favore barri quale decisione relativa alle possibilità di trattamento è stata presa durante gli ultimi incontri che lei ha avuto col medico veterinario:

- Eutanasia
- Eutanasia a causa di mancanza di possibili cure
- Proseguimento delle cure mediche
- Cure palliative
- Interruzione delle cure in attesa della morte naturale
- Rimanere in attesa della morte naturale a causa della mancanza di possibili cure
- Altro

	**non è affatto così**	**non è del tutto così**	**più no che sì**	**è più o meno così**	**è quasi totalmente così**	**è totalmente così**
1. Il mio medico veterinario mi ha comunicato chiaramente che doveva essere presa una decisione.	◯	◯	◯	◯	◯	◯
2. Il mio medico veterinario voleva sapere da me esattamente come volessi partecipare alla decisione.	◯	◯	◯	◯	◯	◯
3. Il mio medico veterinario mi ha comunicato che esistevano diverse possibilità di trattamento per i disturbi del mio animale da compagnia.	◯	◯	◯	◯	◯	◯
4. Il mio medico veterinario mi ha spiegato accuratamente vantaggi e svantaggi delle diverse possibilità di trattamento.	◯	◯	◯	◯	◯	◯
5. Il mio medico veterinario mi ha aiutato a capire tutte le informazioni.	◯	◯	◯	◯	◯	◯
6. Il mio medico veterinario mi ha chiesto quale possibile trattamento preferissi.	◯	◯	◯	◯	◯	◯
7. Il mio medico veterinario ed io abbiamo valutato accuratamente le diverse possibilità di trattamento.	◯	◯	◯	◯	◯	◯
8. Il mio medico veterinario ed io abbiamo scelto insieme un trattamento.	◯	◯	◯	◯	◯	◯
9. Il mio medico veterinario ed io abbiamo trovato un accordo su come procedere in seguito.	◯	◯	◯	◯	◯	◯

### Appendix A.3. Pet Bereavement Questionnaire PBQ

Le chiediamo di esprimere il suo grado di accordo/disaccordo con le seguenti affermazioni secondo la seguente scala di risposte:

0 = per niente d’accordo 1 = poco d’accordo 2 = abbastanza d’accordo 3 = molto d’accordo

1. Mi sento arrabbiato/a con il veterinario per non essere stato in grado di salvare il mio animale da compagnia.	**0**	**1**	**2**	**3**
2. Sono molto turbato/a per la morte del mio animale da compagnia.	0	1	2	3
3. La mia vita sembra vuota senza il mio animale da compagnia.	0	1	2	3
4. Ho avuto incubi sulla morte del mio animale da compagnia.	0	1	2	3
5. Mi sento solo/a senza il mio animale da compagnia.	0	1	2	3
6. Avrei dovuto sapere che sarebbe potuto succedere qualcosa di brutto al mio animale da compagnia.	0	1	2	3
7. Mi manca enormemente il mio animale da compagnia.	0	1	2	3
8. Mi sento molto in colpa per non essermi preso/a più cura del mio animale da compagnia.	0	1	2	3
9. Sto male per non aver fatto di più per salvare il mio animale da compagnia.	0	1	2	3
10. Piango quando penso al mio animale da compagnia.	0	1	2	3
11. Sono arrabbiato/a con altre persone per aver contribuito alla morte del mio animale da compagnia.	0	1	2	3
12. Sono molto triste per la morte del mio animale da compagnia.	0	1	2	3
13. Sono arrabbiato/a con i miei amici/familiari per non essere stati di maggior aiuto.	0	1	2	3
14. I ricordi degli ultimi momenti di vita del mio animale da compagnia mi perseguitano.	0	1	2	3
15. Non supererò mai la perdita del mio animale da compagnia.	0	1	2	3
16. Vorrei aver dimostrato più amore al mio animale da compagnia.	0	1	2	3

### Appendix A.4. Regret of Bereaved Family Members (RBFM)

Le chiediamo di pensare alle decisioni prese per il suo animale da compagnia nelle ultime fasi di vita del suo animale e in particolare alla decisione presa relativamente alla sua morte.

Indichi per ogni affermazione il suo grado di accordo/disaccordo su una scala da 1 a 5.

1 = totalmente in disaccordo 2 = abbastanza in disaccordo 3 = nè in disaccordo, nè in accordo

4 = abbastanza d’accordo 5 = totalmente d’accordo

1. Una volta che comincio a pensare alle possibili conseguenze se avessi preso una decisione diversa, trovo difficile pensare ad altre cose.	**1**	**2**	**3**	**4**	**5**
2. Ho difficoltà a concentrarmi sulle attività quotidiane perché pensieri di rimorso/rimpianto continuano a entrarmi in testa.	1	2	3	4	5
3. Non riesco a smettere di pensare che la situazione sarebbe potuta cambiare se avessi preso una decisone diversa.	1	2	3	4	5
4. E’ stata la decisione giusta.	1	2	3	4	5
5. Prenderei la stessa decisione se dovessi rifarlo.	1	2	3	4	5
6. Mi pento della decisione che è stata presa.	1	2	3	4	5
7. Sono soddisfatto della decisione.	1	2	3	4	5
